# In vivo animal models confirm an increased virulence potential and pathogenicity of the NAP1/RT027/ST01 genotype within the *Clostridium difficile* MLST Clade 2

**DOI:** 10.1186/s13099-020-00383-4

**Published:** 2020-09-22

**Authors:** Josué Orozco-Aguilar, Alejandro Alfaro-Alarcón, Luis Acuña-Amador, Esteban Chaves-Olarte, César Rodríguez, Carlos Quesada-Gómez

**Affiliations:** 1 Laboratorio de Ensayos Biológicos (LEBi), Universidad de Costa Rica, San José, Costa Rica; 2 Facultad de Farmacia, Universidad de Costa Rica, San José, Costa Rica; 3 Programa de Posgrado en Microbiología, Parasitología, Química Clínica e Inmunología, Universidad de Costa Rica, San José, Costa Rica; 4 Departamento de Patología, Escuela de Medicina Veterinaria, Universidad Nacional, Heredia, Costa Rica; 5 Centro de Investigación en Enfermedades Tropicales and Facultad de Microbiología, Universidad de Costa Rica, San José, Costa Rica

**Keywords:** Pathogenicity, Virulence, *Clostridium difficile*, MLST Clade 2, NAP1/RT027/ST01

## Abstract

**Background:**

Based on MLST analyses the global population of *C. difficile* is distributed in eight clades, of which Clade 2 includes the “hypervirulent” NAP1/RT027/ST01 strain along with various unexplored sequence types (STs).

**Methods:**

To clarify whether this clinically relevant phenotype is a widespread feature of *C. difficile* Clade 2, we used the murine ileal loop model to compare the in vivo pro-inflammatory (TNF-α, IL-1β, IL-6) and oxidative stress activities (MPO) of five Clade 2 clinical *C. difficile* isolates from sequence types (STs) 01, 41, 67, and 252. Besides, we infected Golden Syrian hamsters with spores from these strains to determine their lethality, and obtain a histological evaluation of tissue damage, WBC counts, and serum injury biomarkers (LDH, ALT, AST, albumin, BUN, creatinine, Na^+^, and Cl^−^). Genomic distances were calculated using Mash and FastANI to explore whether the responses were dictated by phylogeny.

**Results:**

The ST01 isolate tested ranked first in all assays, as it induced the highest overall levels of pro-inflammatory cytokines, MPO activity, epithelial damage, biochemical markers, and mortality measured in both animal models. Statistically indistinguishable or rather similar outputs were obtained for a ST67 isolate in tests such as tissue damage, neutrophils count, and lethal activity. The results recorded for the two ST41 isolates tested were of intermediate magnitude and the ST252 isolate displayed the lowest pathogenic potential in all animal experiments. This ordering matched the genomic distance of the ST01 isolate to the non-ST01 isolates.

**Conclusions:**

Despite their close phylogenic relatedness, our results demonstrate differences in pathogenicity and virulence levels in Clade 2 *C. difficile* strains, confirm the high severity of infections caused by the NAP1/RT027/ST01 strain, and highlight the importance of *C. difficile* typing.

## Introduction

The main causative agent of nosocomial diarrhea associated with the use and abuse of antibiotics is *Clostridium difficile*; an anaerobic, Gram-positive, spore-forming bacterium. *C. difficile* infection (CDI) symptoms include watery diarrhea, anorexia and leukocytosis and in severe disease presentations these features could be accompanied by dehydration, hypoalbuminemia, acute kidney injury, and hypotension or death [1]. Different genotypes of this bacterium have been isolated worldwide and the prevalence rates varied between the countries [2].

Most *C. difficile* strains produce toxin A (TcdA) and/or toxin B (TcdB), which display cytotoxic activity through glycosylation of small, monomeric GTPases. In addition, a subset of strains synthesize an actin-ADP-ribosylating toxin known as binary toxin (CDT) that targets the actin cytoskeleton [3]. Genes encoding TcdA and TcdB are located on a so-called Pathogenicity Locus (PaLoc). The genes for CDT, by contrast, are typically located elsewhere on the chromosome [4] or on extrachromosomal molecules [5, 6].

TcdA and TcdB induce the secretion of several proinflammatory cytokines (e.g. IL-1β, IL-12, and TNF-α) and lead to neutrophil and macrophage infiltration to intestinal tissues that can result in host damage, as seen in intestinal histopathology specimens [7, 8].

The global population of *C. difficile* is distributed in eight multilocus sequence typing (MLST) clades, of which five contain strains of common detection in human hosts [9]. In the last decade, many countries have reported outbreaks of CDI, partly due to the emergence and rapid spread of hypervirulent or epidemic strains, such as one classified as NAP1 by PFGE, RT027 by ribotyping, and ST01 by MLST [10–13].

Besides its epidemic potential, the NAP1/RT027/ST01 strain has received particular attention due to its link to CDI cases of increased severity and mortality [14]. This strain overproduces TcdA and TcdB, secretes binary toxin (CDT), harbors mutations in genes encoding S-layer proteins (SLPs) that increase its adherence to the gut epithelium and overproduce spores [15]. Some reports have highlighted these characteristics as likely contributors to its increased virulence [10]. However, the magnitude of these phenotypes is not equal in all isolates from this genotype [16], and their relative contribution to its epidemicity remains to be elucidated.

The NAP1/RT027/ST01 strain is classified into the so-called hypervirulent MLST Clade 2 [17, 18] along with 66 additional STs [19, 20]. It is yet unknown whether “hypervirulence” is a universal trait of the members of this clade. To answer this open question and to widen our current knowledge on the pathogenesis of *C. difficile* Clade 2 strains, we performed a comprehensive in vivo analysis of the pathogenicity and lethality of clinical *C. difficile* strains typed as ST01, ST41, ST67, and ST252 in two animal models and interpreted the results on the basis of their phylogenomic relatedness.

## Results

### 5758-ST01 and 5757-ST67 bacteria-free supernatants induced the strongest levels of pro-inflammatory response and tissue damage in the murine ileal loop model

**Fig. 1:**
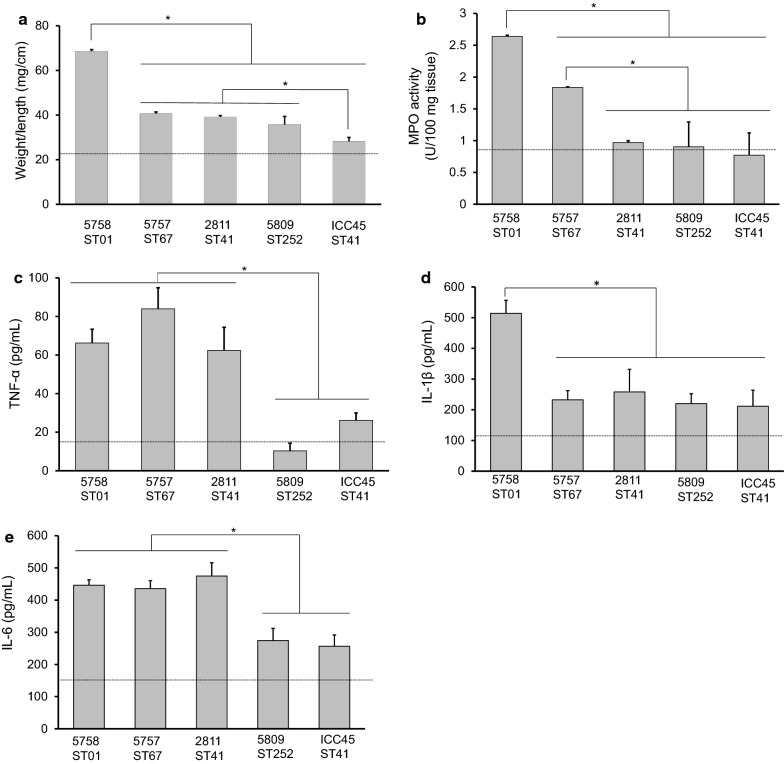
Effect of bacterial cell-free supernatants of Clade 2 *C. difficile* strains in the ligated murine ileal loop. Bacteria-free supernatants from the indicated genotypes were prepared in TYT-broth. 7–10 mice per group were inoculated with 0.3 ml of supernatant in ligated ileal loops. Four hours after inoculation, mice were euthanized and data was recorded. **a** ratio weight/length, bars represent means of the normalized weight of ileal sections. **b** myeloperoxidase activity (MPO) for each group. Cytokines levels were measured on intestinal tissue. **c** TNF-α, **d** IL-1β, and **e** IL-6. *p <0.05 One-way ANOVA followed by Bonferroni’s correction. The mean of measurements obtained for control groups (PBS, TYT-broth and non-toxigenic supernatant) is indicated by a dashed line

The pathogenic potential of the strains was assessed through inoculation of bacterial cell-free supernatants in murine ileal loops and subsequent measurement of the normalized weight of ileal sections as a proxy for edema, MPO (myeloperoxidase) activity as an indicator of neutrophilic degranulation, and several cytokines as markers of acute inflammation. A normalization of supernatant volumes was not required because all the supernatants were taken at equivalent time points of the growth curve. Supernatants from strain 5758-ST01 induced the highest level of edema (69 mg/cm, *p* < 0.05, Fig. 1a). Strains 5757-ST67, 2811-ST41, and 5809-ST252 induced intermediate responses (39–41 mg/cm), which in all cases were above the levels determined for ICC45-ST41 (28 mg/cm) and the controls (PBS, TYT broth, or non-toxigenic *C. difficile* ATCC^®^ 700057 supernatant, 24 mg/cm) (Fig. 1a).

As to MPO activity, 5758-ST01 and 5757-ST67 supernatants caused a significant increase (2.6 and 1.8 U/100 mg of tissue) in its activity compared to supernatants from the other isolates with the former strain causing the highest MPO release overall (*p* < 0.05) (Fig. 1b).

**Fig. 2:**
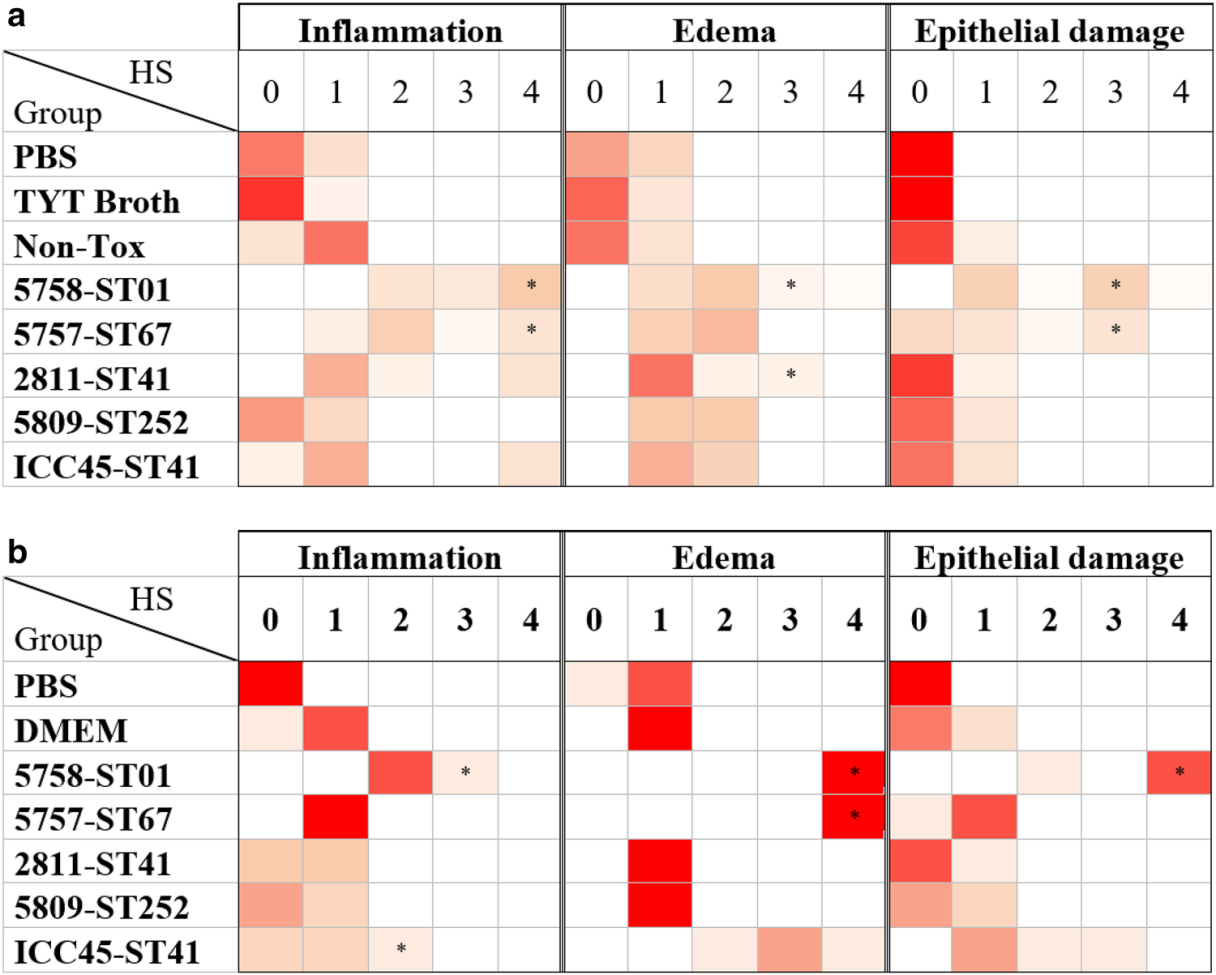
Histopathological scores obtained from intestinal tissue inoculated with bacteria-free supernatants in the murine ligated ileal loop model and of cecal tissue from infected hamsters. **a** Ligated-ileal loop in 7–10 mice per group were inoculated with bacteria-free supernatants of the indicated genotypes. Four hours after treatment the animals were euthanized and intestinal tissues were collected and processed as described in the Methods section. Tissue alterations were scored on coded slides, using a histopathological score (HS) scale from 0 (absence of alterations) to 4 (severe) for three different effects: inflammation (neutrophil infiltration), edema, and epithelial damage. **b** Syrian Golden hamsters previously treated with clindamycin were orally inoculated with spores from the indicated strains. A chirurgical resection of the cecum of the deceased individuals was performed to record histopathological damage. These alterations were scored on coded slides, using a histopathological score (HS) scale that ranges from 0 (absence of alterations) to 4 (severe) for three different effects: inflammation (neutrophil infiltration), edema, and epithelial damage. Results are presented using a heatmap with a scale that ranges from red (100% of samples) to white (0% of samples). *p < 0.05 Kruskall-Wallis and Dunn’s multiple comparison test with Benjamini–Hochberg correction

Regarding the detection of cytokines in ileal tissue, 5758-ST01, 5757-ST67, and 2811-ST41 supernatants induced an increase in TNF-α (62–83 pg/mL) and IL-6 (446–475 pg/mL) levels when compared with supernatants from the other strains (256–274 pg/mL). Additionally, 5758–ST01 induced a higher level of IL-1β (514 pg/mL, *p* < 0.05) (Fig. 1c, d, e). Finally, and in line with the results described above, the histopathologic analyses revealed a greater level of cellular infiltration and epithelial damage in tissue exposed to supernatants from 5758-ST01 or 5757-ST67 (Fig. 2a and Additional file 1: Fig. S1).

### 5758-ST01 strain was more lethal, caused more epithelial destruction, and a stronger pro-inflammatory response in the hamster model

Clindamycin-treated hamsters were administered with *C. difficile* spores and their survival rate was measured daily for 15 days. Whereas 5758-ST01 strains colonized the animals within 1 to 3 days, the remaining strains required 3 to 5 days to achieve the same output. Hamsters infected with 5758-ST01 spores showed wet-tail 1.5 days post-inoculation. A similar result was recorded for animals that received spores from strains 5757-ST67 or 2811-ST41, as they developed wet-tail after 2 to 3 days. This sign was not observed after 5 days for the other strains and the controls.

**Fig. 3:**
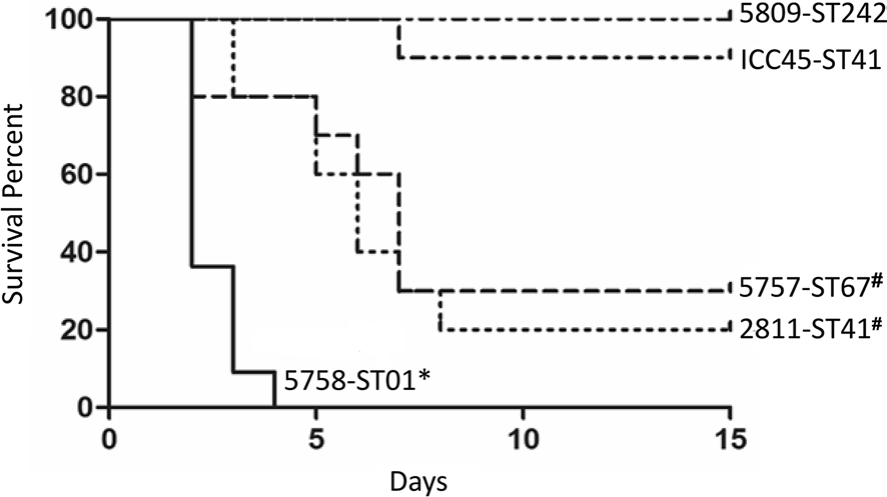
Kaplan-Meier survival curves of hamsters infected with spores of strains from different STs within the *C. difficile* Clade 2. Syrian Golden hamsters previously treated with clindamycin were orally inoculated with spores from the indicated strains. Hamsters were monitored at 12 h intervals for signs of *C. difficile* infection, and the numbers of deceased animals were recorded. *C. difficile* isolates obtained from fecal pellets were typed to confirm the inoculated strain. *^,#^*p* < 0.05 Mantel-Cox test

The survival rates of hamsters inoculated with 5758-ST01 decreased rapidly and none of them survived by day 4 (Fig. 3). By contrast, hamsters infected with 5757- ST67 or 2811-ST41 spores reached 30% survival at day 7. Hamsters inoculated with spores from strains ICC45-ST41 or 5809-ST252 presented survival rates of 90% and 100% by day 12, respectively (Fig. 3).

In agreement with these results, the histopathologic evaluation showed an increased cell infiltration and epithelial damage in the cecum of hamsters infected with 5758-ST01 when compared to the other strains (*p* < 0.5, Fig. 2b and Additional file 1: Fig. S2). Furthermore, the edema produced by 5758-ST01 was similar to the one measured in 5757-ST67 infected animals (HS = 4).

### Strains 5758-ST01 and 5757-ST67 triggered an increase in neutrophil counts and biochemical alterations in peripheric blood in hamsters

**Fig. 4:**
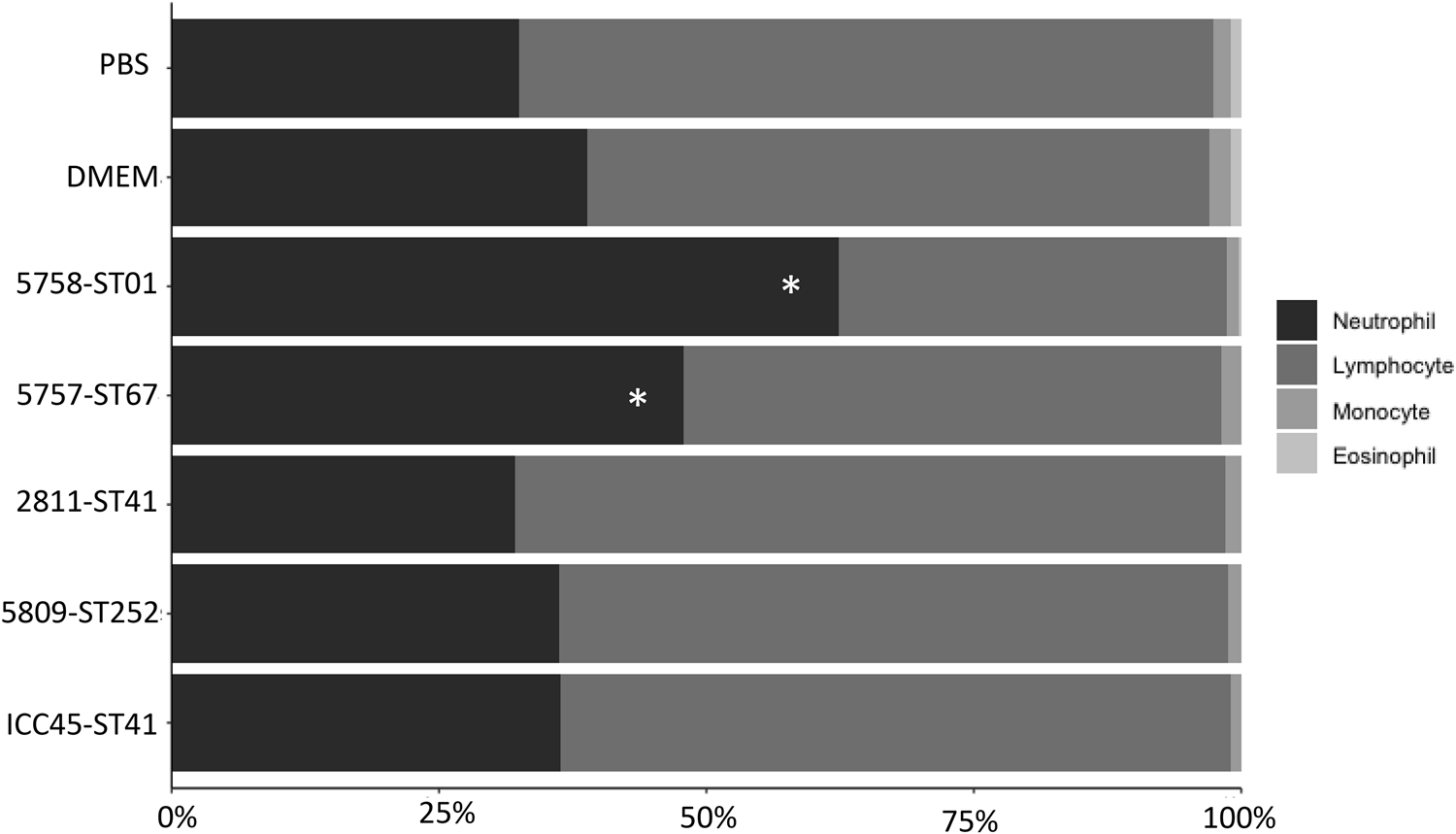
White blood cell (CBC) counts in infected hamsters measured 1 or 2 days after *C. difficile* detection in their feces. Animals were euthanized on day 6 after infection (those with 5758-ST01 were euthanized on day 3 due to their rapid mortality and detection of *C. difficile* in the feces at this time point). Blood samples were collected and WBC counts were performed on stained smears. Counts are expressed in percentages of cells observed. *p < 0.05 Kruskall-Wallis and Dunn’s multiple comparison test with Benjamini–Hochberg correction

After 1 or 2 days of a *C. difficile* positive feces culture of all animals from each group the euthanasia was performed, then white blood cells (WBC) differential and blood biochemical tests were obtained. Compared to the other groups, hamsters inoculated with strains 5758-ST01 or 5757-ST67 showed signs of infection, such as an increase in peripheral neutrophils (50–60%, *p* < 0.5) (Fig. 4).

**Fig. 5:**
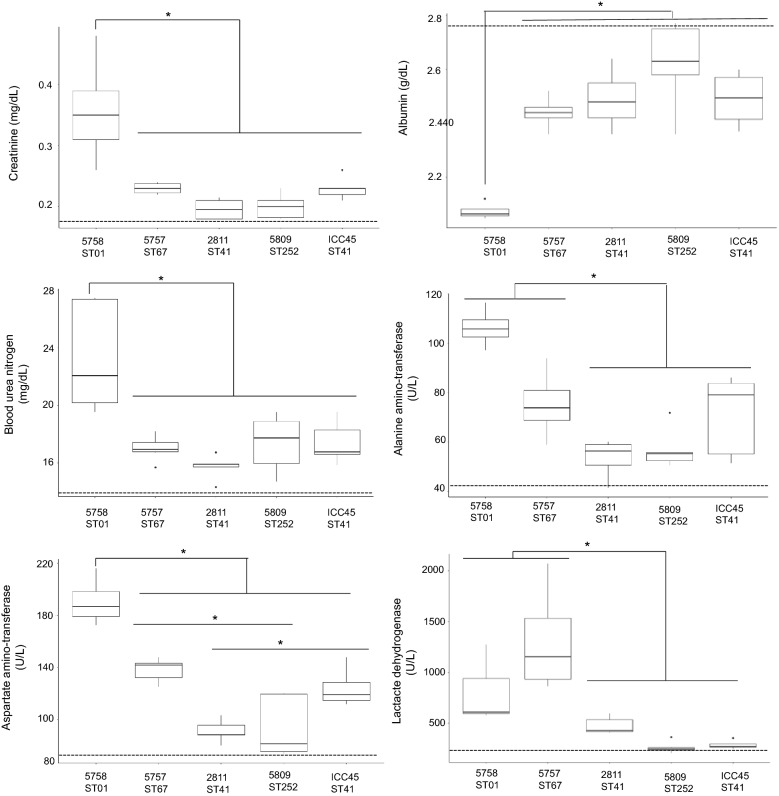
Serological parameters of infected hamsters measured 1 or 2 days after *C. difficile* detection in their feces. Animals were euthanized 1 or 2 days after *C. difficile* detection on feces (day 3 for 5758-ST01 and day 6 for all other groups). The results are presented as the average ± standard error. The following biomarkers were measured in hamster serum: **a** creatinine, **b** blood urea nitrogen (BUN), **c** alanine amino-transferase (ALT), **d** aspartate amino-transferase (AST), **e** lactate dehydrogenase (LDH), and **f** albumin. * *p* < 0.05 One-way ANOVA with Bonferroni’s correction. The mean of measurements for control groups (DMEM and PBS) is indicated by a dashed line

The biochemical testing suggested a general multiorgan alteration in hamsters infected with 5758-ST01. Unlike other groups, animals that received 5758-ST01 spores presented alterations in liver enzymes (ALT and AST), serum proteins synthesized in the liver (albumin), renal function impairment (creatinine and BUN), tissue damage (LDH), and serum electrolytes (sodium and chloride) (*p* < 0.5) (Fig. 5). Animals infected with 5757-ST067 showed similar alterations in ALT and LDH as hamsters that received 5758-ST01 spores (*p* < 0.5). The biochemical analytes measured in all other animals were indistinguishable from those recorded in the control groups that received PBS or DMEM (*p* > 0.5) (Fig. 5).

### 5757-ST67 showed the smallest genomic distance of all tested strains to 5758-ST01

The magnitude of the in vivo phenotypes matched their genomic distance of all non-ST01 isolates to 5758-ST01. The smallest Mash distance (0.0048) and highest ANI value (99.34) was determined for strain 5757-ST67, which was followed by the two ST41 strains tested (2811-ST41 and ICC45-ST41, Mash = 0.0061–0.0062, ANI = 99.18-99.30) and finally by strain 5809-ST252 (Mash = 0.0096, ANI = 98.86). (Table 1).

**Table 1:** Genomic relatedness of Clade 2 *C. difficile* strains compared to strain 5758-ST01

Strain	ANI^a^	Mash^b^
5757-ST67	99.34 (1296/1354)	0.0048 (826/1000)
ICC45-ST41	99.30 (1313/1427)	0.0061 (786/1000)
2811-ST41	99.18 (1296/1402)	0.0062 (781/1000)
5809-ST252	98.86 (1262/1381)	0.0096 (690/1000)

^a^ Average nucleotide identity (ANI) values (bidirectional fragment mapping/total query fragments)

^b^ Mash distance (shared-hashes)

## Discussion

The use of MLST technique has facilitated the classification of isolates into several clades. Clade 2 includes ST01 strains [17, 18], which have caused outbreaks worldwide [21, 22]. This clade has been divided into subgroups 2i (ST01, ST67, ST41, ST62 or ST140) and 2ii (ST32, ST47, ST61, ST95, ST97, ST123 or ST252) [23]. This work revealed a broad spectrum of virulence and pathogenic potential among five clinical isolates from different STs within the *C. difficile* “hypervirulent” Clade 2.

Strain 5758-ST01 from subgroup 2i was the most virulent strain in all in vivo experiments, as it induced higher cytokine secretion in intestinal tissue, tissue damage, multiorgan alterations, and mortality. This could be due a combination of already reported characteristics of the NAP1/RT027/ST01 strain, such as toxin overproduction [24], binary toxin secretion [25], and increased sporulation [26]. However, other unexplored mechanisms such as polymorphisms and/or regulation patterns of colonization determinants like S-layer protein, flagellin, β-glucosidases and other adhesins [27–30] could also have played a role in the pathogenesis. A detailed studied of those mechanisms is beyond the scope of this study.

An even wider pathogenicity spectrum could have been observed if more isolates from other STs had been studied. Nonetheless, our results suggest that the relative virulence of the non-ST01 strains goes in line with their phylogenomic distance to the well-characterized NAP1/RT027/ST01 genotype.

Strain 5757-ST67 was second in terms of the damage inflicted in animal experiments. This is somewhat expectable, as ST-67 strains also belong to subgroup 2i and have been reported to cause severe CDI [31]. Despite having a variant TcdB, 5757-ST67 produced an elevated inflammatory response and alteration of biochemical markers that could be explained by toxin overproduction [32]. Moreover, mutations in *treR* (trehalose repressor), CpG depleted genomes [31] and a PaLoc rather similar to that of strain R202091-ST01 [33] have been described in this ST, all of which are characteristics of hypervirulent *C. difficile* strains.

The proinflammatory response and mortality levels recorded for strain 2811-ST41, also belonging to subgroup 2i, did not reach 5758-ST01 and 5757-ST67 levels. Furthermore, strain ICC45-ST41 produced weaker proinflammatory responses than 2811-ST41. Among multiple explanations, this difference could be tracked to the synthesis of a variant TcdB in the former [34]. However, information on the amount of secreted TcdA and CDT by strains from this ST is still lacking, though it could clarify the observed differences in proinflammatory potential.

Strain 5809-ST252, from subgroup 2ii, presented the weakest proinflammatory response and lethal potential of the studied strains. We did not find reports in the literature on the pathogenesis of this type of *C. difficile* strains and therefore cannot provide explanations for its behavior.

Our results could be explained by undescribed differences in the strains´ exoproteomes and attributed to structural particularities on the bacterial surface that alter human intestinal homeostasis [29]. Other factors, unexplored in this work, such as germination potential, colonization efficiency, the number of metabolically active *C. difficile* cells in the intestine, and bile acids metabolism could explain the differences in the damage observed in the infection models [35–37].

Contradictory observations on in vitro phenotypes expressed by isolates from the same ribotype, such as RT027, have been reported, particularly with regard to sporulation and toxin production [16, 38, 39]. Therefore, in vivo studies, such as the work herein presented, have the potential to clarify pending issues and should be carried out with two or more animal models and including others STs from the “hypervirulent” Clade 2.

We observed indications of extraintestinal organic damage, mainly in renal and hepatic functions, as well as an increase in leukocytes, these alterations were recorded after 1 or 2 days of *C. difficile* detection on feces. The animals infected with 5758-ST01 showed altered biochemical parameters sooner than those infected with non-ST01 strains (exposed for 3 additional days). As such, in an early infection, the general health condition is altered by 5758-ST01 infection but not by non-ST01 strains. These findings have been described in the severe outcome of CDI in humans [40, 41], which suggest a severe pathology in animals infected by the 5758-ST01 strain.

We conclude that “hypervirulence” is not a widespread feature in *C. difficile* Clade 2 strains. This result lay the foundations for interpretation of future in vitro and genomic comparisons of these and other Clade 2 strains. Moreover, it highlights the importance of *C. difficile* typing and of targeted diagnosis of NAP1/027/ST01 strains.

## Materials and methods

### *C. difficile* strains

This study was done with five *C. difficile* MLST Clade 2 strains that were isolated from stool samples of symptomatic patients. Briefly, samples were treated with 96% ethanol and inoculated onto Cefoxitin-Cycloserine-Fructose agar plates (Oxoid) that were later incubated in anaerobic chamber (90% N_2_, 5% CO_2_, 5% H_2_). Colonies were identified phenotypically with the RapID 32A system (bioMerieux) and by PCR-amplification of the *tpi* gene. These bacteria were previously analyzed by PFGE, an end-point PCR targeting different PaLoc fragments, and toxigenic culture following the previously reported protocols [42]. All strains were cryopreserved at − 80° C and recovered through culturing on Brucella agar plates supplemented with vitamin K and laked horse blood under anaerobic conditions (Table 2).

**Table 2:** *C. difficile* MLST Clade 2 strains used in this study

Strain	ST	PFGE-type^a^	PCR PaLoc profile	CPE^b^
5758	01	NAP1	*tcdA*^+^/*tcdB*^+^/*cdtB*^+^/*tcdC*^+, del^	C^Ov^
2811	41	New type	*tcdA*^+^/*tcdB*^+^/*cdtB*^+^/*tcdC*^+^	C
ICC45	41	New type	*tcdA*^+^/*tcdB*^+^/*cdtB*^+^/*tcdC*^+^	V
5757	67	NAP1	*tcdA*^+^/*tcdB*^+^/*cdtB*^+^/*tcdC*^+, del^	V^Ov^
5809	252	New type	*tcdA*^+^/*tcdB*^+^/*cdtB*^+^/*tcdC*^+^	C

^a^ According to the database of the National Microbiology Laboratory of Canada

^b^ CPE, cytopathic effect; C, classic; V, variant [32, 34]

^del^ 18 pb deletion in *tcdC*

^Ov^ Toxin overproduction [32, 34]

### Preparation of bacterial cell-free supernatants

All strains were grown in TYT-broth (3% tryptose, 2% yeast extract, 0.1% thioglycolate, and pH 6.8) for 24 h under anaerobic conditions. At this time point, viable bacterial cells counts for all strains were in the 10^7^ CFU/mL order (Additional file 1: Fig. S3). Bacterial cells were then pelleted by centrifugation for 30 min at 20,000 g and the resulting supernatants were filtered using 0.2 µm pore membranes [32]. These bacterial cell-free supernatants were used in the ileal loop assay in mice (see below).

### Murine ileal loop model

Four to 5 weeks old, male, Hsd:ICR mice with a body weight of 18–25 g were used. These animals were grouped in polycarbonate cages and maintained under controlled conditions of temperature (19.9 ± 0.7 °C and 23.5 ± 1.0 °C), relative moisture (75 ± 5% and 89 ± 3%), noise (81.4 ± 2.2 dB); and photoperiod (12 h). Water and feed were available ad libitum. All animal proceedings were in compliance with local legislation (Ley de Bienestar de los Animales N° 7451) and approved by the Comité Institucional de Cuido y Uso de Animales (CICUA) from the Universidad de Costa Rica (CICUA 52-15). We used the maximum amount of animals authorized by CICUA based on the 3R’s principles.

Mice were fasted overnight and then anesthetized with ketamine (60 mg/kg) and xylazine (5 mg/kg). Through a midline laparotomy, a 4 cm ileal loop was ligated and injected with 0.3 ml of bacterial cell-free supernatants or control solutions (PBS, TYT-broth or non-toxigenic *C. difficile* ATCC^®^ 700057 supernatant). Four hours after inoculation mice were euthanized and the length and weight of the intestinal loops were recorded [43, 44].

### Myeloperoxidase (MPO) assay

Neutrophils degranulation in homogenized ileal tissue was evaluated using a colorimetric MPO activity assay [45]. Briefly, ileal tissue (100 mg) was homogenized in hexadecyltrimethylammonium bromide (HTAB) (Sigma) buffer (PBS, HTAB 50% w/v, and H_2_O_2_ 0.1% v/v) and cleared by centrifugation at 4500 g for 7 min at 4 °C. The resulting supernatants were incubated with a 0.017% *o*-dianisidine solution (Sigma) and after 5 min the absorbance was determined at 450 nm. Results were reported as MPO/100 mg of ileal tissue.

### Detection of TNF-α, IL-1β, IL-6, and IL-10

The concentration of proinflammatory interleukins (IL-1β, IL-6, IL-10) and tumor necrosis factor alpha (TNF-α) in ileal tissue homogenates was determined with commercial enzyme-linked immunosorbent assays (ELISA) following the instructions of the manufacturer (R&D Systems).

### Hamster infection model

Six to eight weeks old, male, Golden Syrian hamsters (*Mesocricetus auratus*) with a body weight of 85–120 g were used. Similarly to previous studies [46, 47], the hamsters were separated into groups of 5 animals.

The animals were maintained in polycarbonate cages under the conditions mentioned above for mice. Two clindamycin doses were administered to each individual through the oral route 10 (30 mg/kg) and 5 days (50 mg/kg) prior to the beginning of the experiment. On day 0, each animal was inoculated orally with 10^4^ spores that were prepared as previously described [48]. Seven groups of animals were used, including five treatments (five strains) and two negative controls (PBS or DMEM).

Animals were monitored for 15 days for infection signs, such as weight loss and wet tail (diarrhea), and to record their mortality. Stool samples were collected every 2 days throughout the experiment for isolation of *C. difficile* following published protocols [49]. When an experimental subject died, or at day 15 (when euthanasia was performed), a chirurgical resection of the ascendant colon and the cecum was performed to monitor colonization by *C. difficile*.

### Analysis of blood biomarkers

Additional groups of 5 *Golden Syrian* hamsters were inoculated with spores of each bacterial strain and negative controls (PBS or DMEM) as described above. All animals infected with 5758-ST01 strain were euthanized on day 3 (due to its rapid lethality rate associated with this strain) and all other groups on day 6 post-spore inoculation. The criteria in this matter was that euthanasia (and subsequent blood collection) should be performed 1 or 2 days after successful colonization or infection by *C. difficile* (as demonstrated by a positive fecal *C. difficile* culture).

Then, a portion of whole blood was collected after decapitation to measure serum levels of albumin, creatinine, blood urea nitrogen (BUN), sodium, chloride, lactate dehydrogenase (LDH), alanine amino-transferase (ALT), and aspartate amino-transferase (AST) levels with an automated analyzer (Roche Cobas^®^ 3c11). Another portion of total blood was collected in microtubes with EDTA to obtain white blood cells (WBC) differential counts through visualization of Wright-stained blood smears.

### Histopathologic assessment of tissue sections

Murine ileal and hamsters’ cecum samples were fixed in 10% buffered formalin, processed, and stained with hematoxylin and eosin (H&E) for histopathological evaluation. These preparations were evaluated for epithelial damage, edema, and neutrophil infiltration using a histopathological scoring (HS) system that ranged from 0 (absence of alterations) to 4 (severe) [50]. All histopathological assays were performed by an expert DVM pathologist in a single blind setting.

### Phylogenomic analyses

Whole genomes were reconstructed from 2 × 250 bp paired-end Illumina reads from the NCBI BioProject PRJNA293889. The genomic relatedness of the non-ST01 isolates to 5757-ST01 strain was determined using Mash [51], which is based on a MinHash dimensionality-reduction technique, and through calculation of average nucleotide identity (ANI) values with an alignment-free method [52].

### Statistical analyses

Data from the animal models are presented as mean ± standard error (SEM) or as medians. Means and median were compared using one-way ANOVA tests with Bonferroni correction or Kruskall-Wallis tests followed by Dunn’s multiple comparison tests with Benjamini–Hochberg adjustment, respectively. Mortality was evaluated using Kaplan–Meier curves. *P* values < 0.05 were considered statistically significant.

## Supplementary information

**Additional file 1: Figure S1.** Histopathological analysis of the effects induced by bacteria-free supernatants in the murine model of ileal ligated loop. Tissue was fixed in 10% buffered formalin and stained with H&E for histological evaluation of the following groups: A) PBS, B) TYT-broth, C) Non-toxigenic *C. difficile* ATCC^®^ 700057, D) 5758-ST01, E) 5757-ST67, F) 2811-ST41, G) 5809-ST252 and H) ICC45-ST41. **Figure S2.** Histopathological analysis of cecum from infected hamsters. Cecum sections were fixed in 10% buffered formalin and stained with H&E for histological evaluation of the following groups: A) PBS, B) DMEM, C) 5758-ST01, D) 5757-ST67, E) 2811-ST41, F) 5809-ST252, and G) ICC45-ST41. **Figure S3.** Counts of viable *C. difficile* cells by strain. Growth curves were performed in TYT-broth under the described conditions. At 0, 8 and 24h, an aliquot was taken, serially diluted and inoculated onto Brucella agar plates supplemented with vitamin K agar plates. The number of colonies that appeared after 48 h of incubation was recorded to obtain CFU/mL values.

## Data Availability

All data generated or analyzed during this study are included in this published article and its Additional file 1.

## Abbreviations

ALT: Alanine amino-transferase
AST: Aspartate amino-transferase
ANI: Average nucleotide identity
BUN: Blood urea nitrogen
CDI: *Clostridium difficile* infection
CDT: Binary toxin
DMEM: Dulbecco’s Modified Eagle’s medium
H&E: Hematoxylin and eosin
HS: Histopathological score
HTAB: Hexadecyltrimethylammonium bromide
IL: Interleukin
LDH: lactate dehydrogenase
MLST: Multilocus sequence typing
MPO: Myeloperoxidase
NAP: North American PFGE type
PaLoc: Pathogenecity locus
PBS: Phosphate buffered saline
RT: Ribotype
SLP: S-layer
ST: Sequence type
TcdA: Toxin A
TcdB: Toxin B
TNF: Tumor necrosis factor
TYT: Tryptose-yeast extract-thioglycolate
WBC: White blood cells
