# Supplementary material for: In vivo animal models confirm an increased virulence potential and pathogenicity of the NAP1/RT027/ST01 genotype within the Clostridium difficile MLST Clade 2
Source: Gut Pathog. 2020 Sep 22;12:45. doi: 10.1186/s13099-020-00383-4 (PMC7510272; doi:10.1186/s13099-020-00383-4)
Supplement: Supplementary file 1 — Additional file 1: Figure S1. Histopathological analysis of the effects induced by bacteria-free supernatants in the murine model of ileal ligated loop. Tissue was fixed in 10% buffered formalin and stained with H&E for histological evaluation of the following groups: A) PBS, B) TYT-broth, C) Non-toxigenic C. difficile ATCC® 700057, D) 5758-ST01, E) 5757-ST67, F) 2811-ST41, G) 5809-ST252 and H) ICC45-ST41. Figure S2. Histopathological analysis of cecum from infected hamsters. Cecum sections were fixed in 10% buffered formalin and stained with H&E for histological evaluation of the following groups: A) PBS, B) DMEM, C) 5758-ST01, D) 5757-ST67, E) 2811-ST41, F) 5809-ST252, and G) ICC45-ST41. Figure S3. Counts of viable C. difficile cells by strain. Growth curves were performed in TYT-broth under the described conditions. At 0, 8 and 24h, an aliquot was taken, serially diluted and inoculated onto Brucella agar plates supplemented with vitamin K agar plates. The number of colonies that appeared after 48 h of incubation was recorded to obtain CFU/mL values. [file 13099_2020_383_MOESM1_ESM.pptx]

## Slide 1
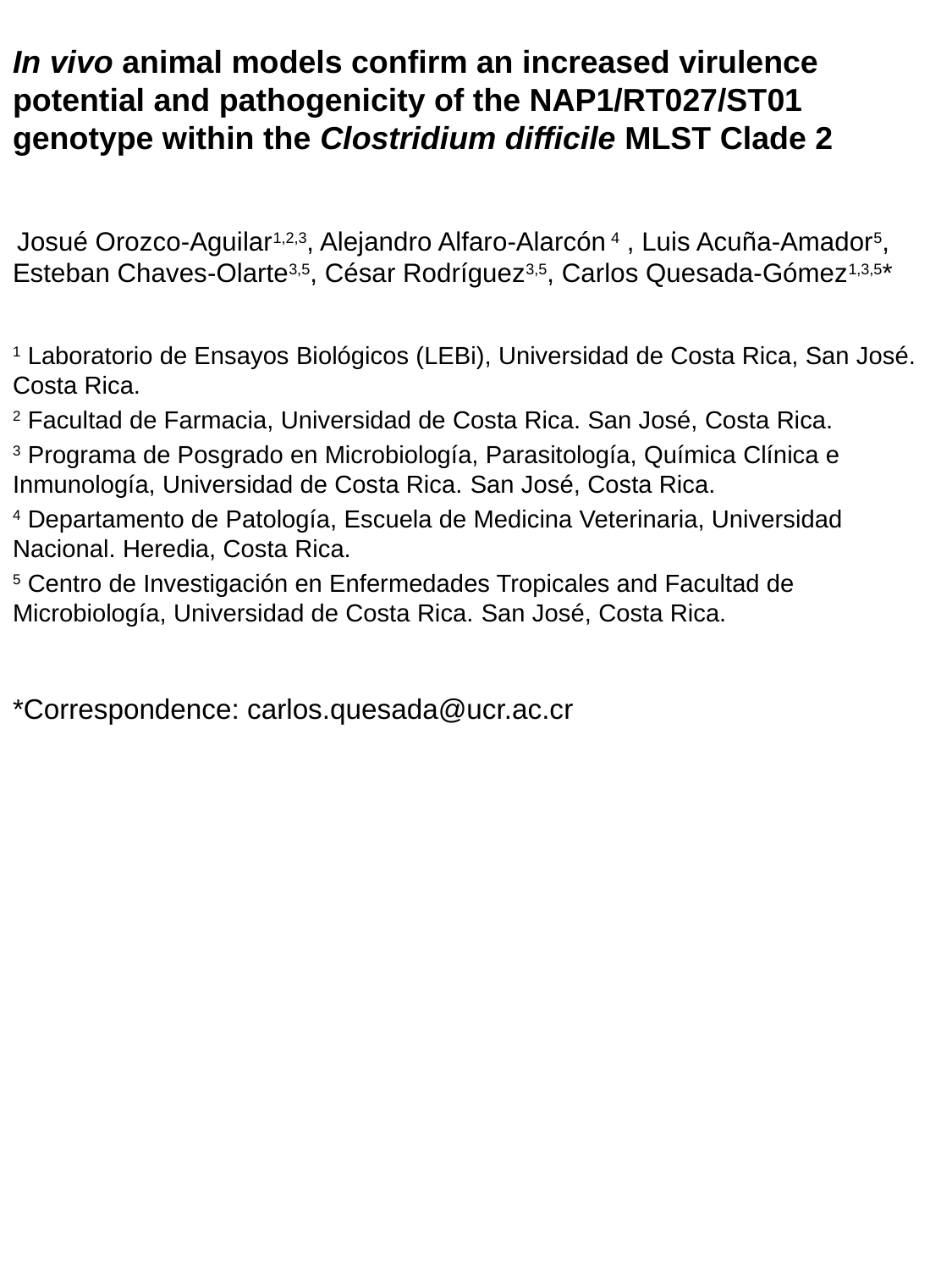

In vivo animal models confirm an increased virulence potential and pathogenicity of the NAP1/RT027/ST01 genotype within the Clostridium difficile MLST Clade 2
 Josué Orozco-Aguilar1,2,3, Alejandro Alfaro-Alarcón 4 , Luis Acuña-Amador5, Esteban Chaves-Olarte3,5, César Rodríguez3,5, Carlos Quesada-Gómez1,3,5*
1 Laboratorio de Ensayos Biológicos (LEBi), Universidad de Costa Rica, San José. Costa Rica.
2 Facultad de Farmacia, Universidad de Costa Rica. San José, Costa Rica.
3 Programa de Posgrado en Microbiología, Parasitología, Química Clínica e Inmunología, Universidad de Costa Rica. San José, Costa Rica.
4 Departamento de Patología, Escuela de Medicina Veterinaria, Universidad Nacional. Heredia, Costa Rica.
5 Centro de Investigación en Enfermedades Tropicales and Facultad de Microbiología, Universidad de Costa Rica. San José, Costa Rica.
*Correspondence: carlos.quesada@ucr.ac.cr

## Slide 2
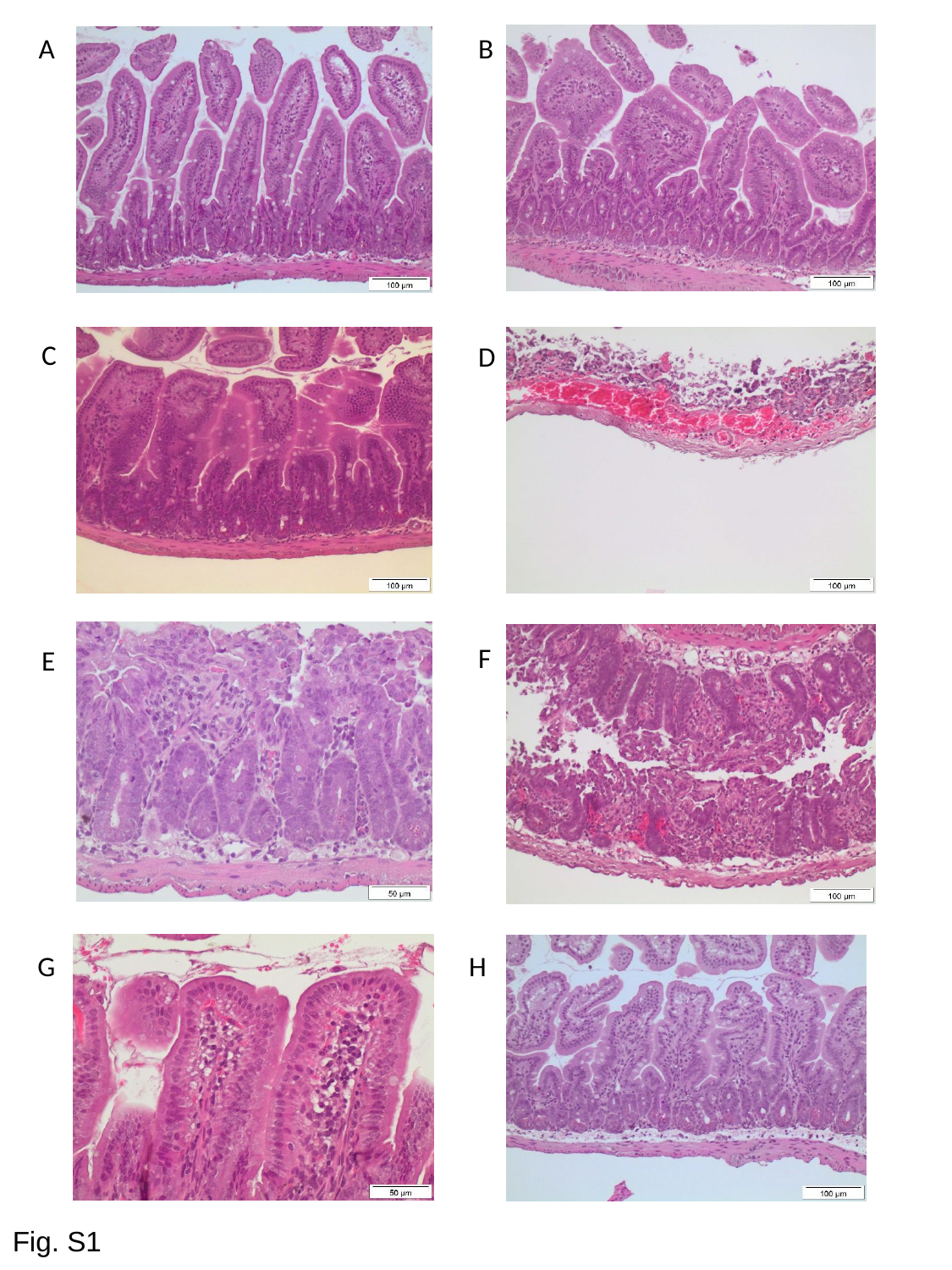

A
B
C
D
F
E
G
H
Fig. S1

## Slide 3
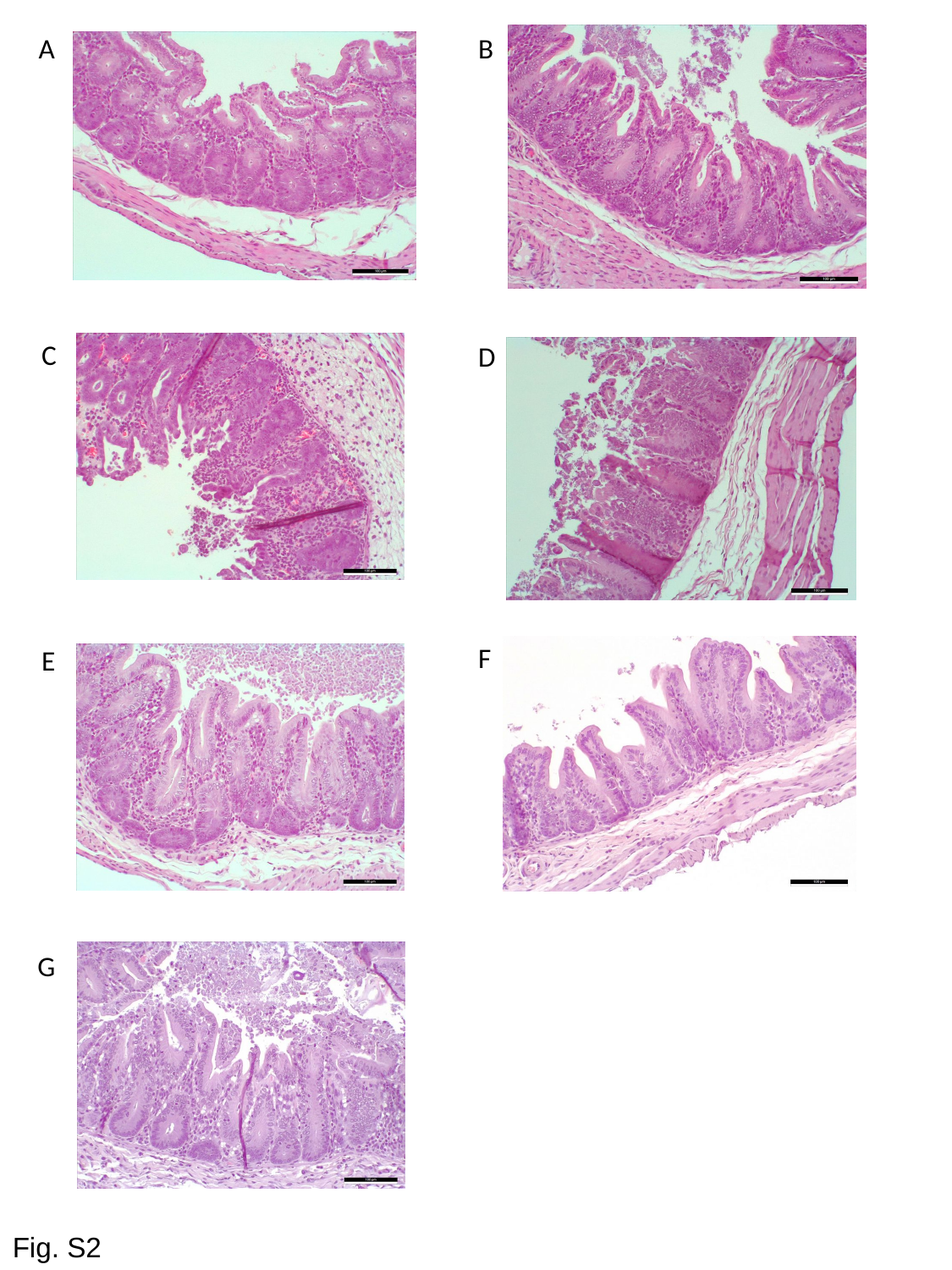

A
B
C
D
F
E
G
Fig. S2

## Slide 4
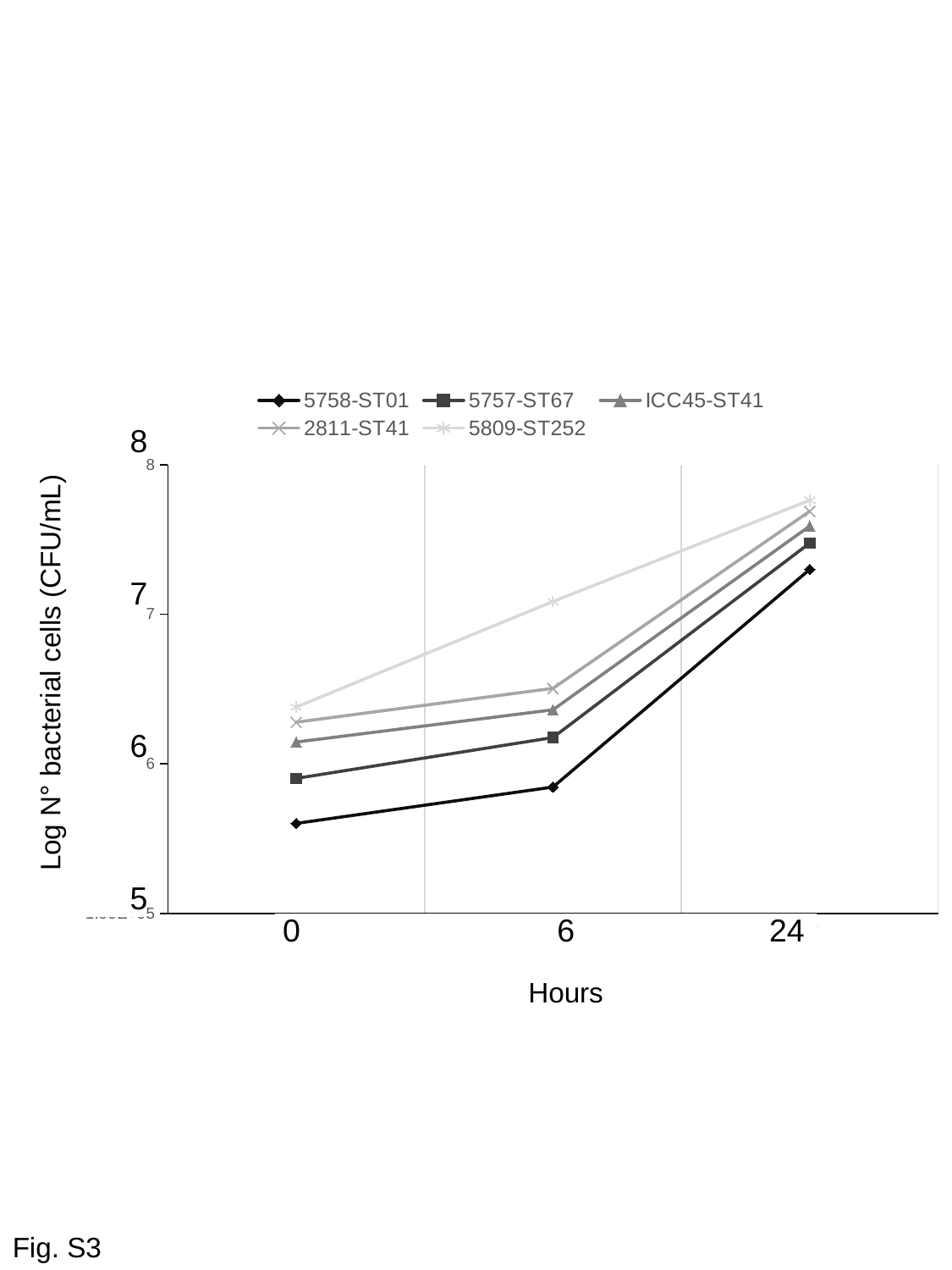

### Chart
| Category | 5758-ST01 | 5757-ST67 | ICC45-ST41 | 2811-ST41 | 5809-ST252 |
|---|---|---|---|---|---|
| 0 | 400000.0 | 400000.0 | 600000.0 | 500000.0 | 500000.0 |
| 6 | 700000.0 | 800000.0 | 800000.0 | 900000.0 | 9000000.0 |
| 24 | 20000000.0 | 10000000.0 | 9000000.0 | 10000000.0 | 9000000.0 |8
7
6
5
Log N° bacterial cells (CFU/mL)
0 6 24
Hours
Fig. S3
